# Prenatal treatment with EGCG enriched green tea extract rescues GAD67 related developmental and cognitive defects in Down syndrome mouse models

**DOI:** 10.1038/s41598-019-40328-9

**Published:** 2019-03-08

**Authors:** Benoit Souchet, Arnaud Duchon, Yuchen Gu, Julien Dairou, Claire Chevalier, Fabrice Daubigney, Valérie Nalesso, Nicole Créau, Yuejin Yu, Nathalie Janel, Yann Herault, Jean Maurice Delabar

**Affiliations:** 1 Université Paris-Diderot, Sorbonne Paris Cité, Adaptive Functional Biology, National Centre for Scientific Research (CNRS), UMR 8251, Paris, France; 2 Institut Génétique Biologie Moléculaire Cellulaire, CNRS, French National Institute of Health and Medical Research (INSERM), UMR 7104, UMR 964, Illkirch, France; 3 Institut de Génétique et de Biologie Moléculaire et Cellulaire, Université de Strasbourg, 1 rue Laurent Fries, 67404 Illkirch, France; 4 CNRS, UMR 7104, Illkirch, France; 5 INSERM, U964 Illkirch, France; 6 Université de Strasbourg, 1 rue Laurent Fries, 67404 Illkirch, France; 7 CNRS, UMR 8601, Laboratoire de Chimie et Biochimie Pharmacologiques et Toxicologiques, Université Paris Descartes-Sorbonne Paris Cité, 75270 Paris, France; 8 Children’s Guild Foundation Down Syndrome Research Program, Department of Cancer Genetics, Roswell Park Cancer Institute, Elm and Carlton Streets, Buffalo, NY 14263 USA; 9 INSERM U 1127, CNRS UMR 7225, Sorbonne Universités, UPMC Univ Paris 06 UMR S 1127, Institut du Cerveau et la Moelle épinière, ICM, Paris, France; 10 Brain & Spine Institute (ICM) CNRS UMR7225, Inserm UMRS 975, Paris, France

## Abstract

Down syndrome is a common genetic disorder caused by trisomy of chromosome 21. Brain development in affected foetuses might be improved through prenatal treatment. One potential target is DYRK1A, a multifunctional kinase encoded by chromosome 21 that, when overexpressed, alters neuronal excitation–inhibition balance and increases GAD67 interneuron density. We used a green tea extract enriched in EGCG to inhibit DYRK1A function only during gestation of transgenic mice overexpressing Dyrk1a (mBACtgDyrk1a). Adult mice treated prenatally displayed reduced levels of inhibitory markers, restored VGAT1/VGLUT1 balance, and rescued density of GAD67 interneurons. Similar results for gabaergic and glutamatergic markers and interneuron density were obtained in Dp(16)1Yey mice, trisomic for 140 chromosome 21 orthologs; thus, prenatal EGCG exhibits efficacy in a more complex DS model. Finally, cognitive and behaviour testing showed that adult Dp(16)1Yey mice treated prenatally had improved novel object recognition memory but do not show improvement with Y maze paradigm. These findings provide empirical support for a prenatal intervention that targets specific neural circuitries.

## Introduction

Typical brains maintain a precise ratio between neuronal excitation and inhibition (E/I) to allow efficient learning. This ratio is established early during neurogenesis. One of the first neurotransmitters to become functional in the developing central nervous system, before functional synapses form, is Υ-aminobutyric acid (GABA)^1^. Indeed, in wildtype mice, one in five migrating neurons at embryonic day E 14 is already established as a GABAergic neuron^2^. GABA is an inhibitory neurotransmitter that regulates E/I balance by binding to GABA_A_ receptors and preventing further signalling from the bound neuron^3^.

E/I balance appears to be altered in the brains of individuals with Down syndrome (DS). Individuals with DS, which results from trisomy for all or part of chromosome 21, exhibit delayed cognitive progress in infancy and childhood, leading to mild to moderate intellectual disability. In contrast to typically-developing brains, it has been hypothesized that the brains of individuals with DS may have too much GABA-related inhibition^4^. In DS foetuses the ratio of calretinin positives cells versus calretinin negative cells was found increased in gyrus, entorhinal cortex and hippocampus^5^. Mouse models of DS also exhibit an imbalanced E/I ratio, with a suppressed hippocampal long-term potentiation^6^ and with an increased level of the glutamic acid decarboxylases GAD65 and GAD67^7^. The observed increase in GADs is similar (130%–150%) both in monogenic models overexpressing only *Dyrk1a*, a serine-threonine kinase implicated in brain development, and in more complex models trisomic for an additional 30–140 chromosome 21 orthologs. Many different approaches have been developed to correct cognitive impairments of DS mice models targeting E/I balance, oxidative stress or other pathways^8^.

The E/I imbalance is present and associated with cognitive impairment in mBACtgDyrk1a (transgenic for a mouse BAC containing the full Dyrk1a gene), hYACtgDyrk1a (trisomic for 5 genes), Ts1Rhr (trisomic for 33 genes), Dp(16)1Yey (trisomic for 140 genes), and Ts65Dn (trisomic for 122 genes orthologous to HSA21 genes and 60 genes not orthologous to HSA21). These DS mouse models^7^ contain Dyrk1a gene in three copies.

Genetic correction of DYRK1A levels in Ts65Dn mice restores a normal phenotype^9^. Partial rescue is also obtained with expression of two copies of *Dyrk1a* in Dp(16)1Yey mice^10^. In comparison with disomic cells, Ts65Dn mouse neural progenitor cells show premature neuronal differentiation and enhanced GABAergic differentiation. Further, treatment with harmine, a competitive inhibitor of DYRK1A, reverses this effect^11^.

Epigallocatechin gallate (EGCG), the major catechin in green tea, is a noncompetitive inhibitor of DYRK1A^12^ with an IC50 of 0.3 µM. Other targets are PRAK (IC50 of 1 µM)^13^ or metallo proteases (IC50 of 20 µM)^14^. It rescues long-term potentiation deficits in CA1-CA3 of Ts65Dn, in neocortex of mBACtgDyrk1a^15^ and molecular and behavioural alterations observed in DS mouse models^16–18^. Clinical trials of a dietary supplement containing EGCG among young adults with DS resulted in a partial correction of cognitive deficits^17,19^. However, mouse models have shown varying results with EGCG treatment. Treatment of adult mice with EGCG for one month does not rescue levels of inhibitory interneuron marker GAD67^18^. EGCG treatment corrects mouse escape latency in the Morris water maze, but not the probe test^17^. Prenatal EGCG treatment modulated trisomic neural crest cells deficiencies at embryonic time points and normalized some craniofacial phenotypes, including cranial vault in adult Ts65Dn mice^20^. Further, EGCG treatment during postnatal days (P) P3–P15 restores neurogenesis at P15 but not at P45, and does not restore normal performance either in Y maze or Morris water maze tests^21^. In contrast, a mouse model transgenic for human DYRK1A treated from gestation to adulthood shows strong correction of molecular (brain-derived neurotrophic factor levels) and cognitive deficits (novel object recognition tests)^16^. In mouse models, *Dyrk1a* is overexpressed as early as E11.5 (telencephalon) and E15.5 (brain)^22,23^. The prenatal period is critical for GABAergic interneurons; therefore, it was relevant to investigate adults for persistence of changes induced by EGCG prenatal treatment.

## Results

### Prenatal EGCG treatment corrects GAD67 level and GAD67 neuron density in mBACtgDyrk1a mice

The decaffeinated green tea extract (MGTE) was evaluated for its *in vitro* DYRK1A inhibitory activity using a fluorescent peptide substrate of this enzyme and UFLC (Ultra Fast Liquid Chromatography) assay as previously described^24^: IC_50_ = 0.35 µM similar to IC_50_ = 0.3 µM found for purified EGCG (see Supplementary Methods). We used EGCG-complemented food pellets (MGTE) corresponding to an intermediate dose of 50 mg/kg, which was determined by a previous dose-effect trial^18^. A first assessment by HPLC experiments (Supplemental Methods, LOD = 25 nM) on wild type animals established that catechins from MGTE extract were found in embryonic (E14) brains (<25 nM), in milk collected from stomachs of P1 and P7 pups (27–57 nM), and in P1 and P7 plasmas (0.5 to 1 µM) in similar amounts as those reported for pharmacokinetics experiments performed in freely moving rats (3 µM in plasma)^25^ (Supp. Methods).

**Figure 1:**
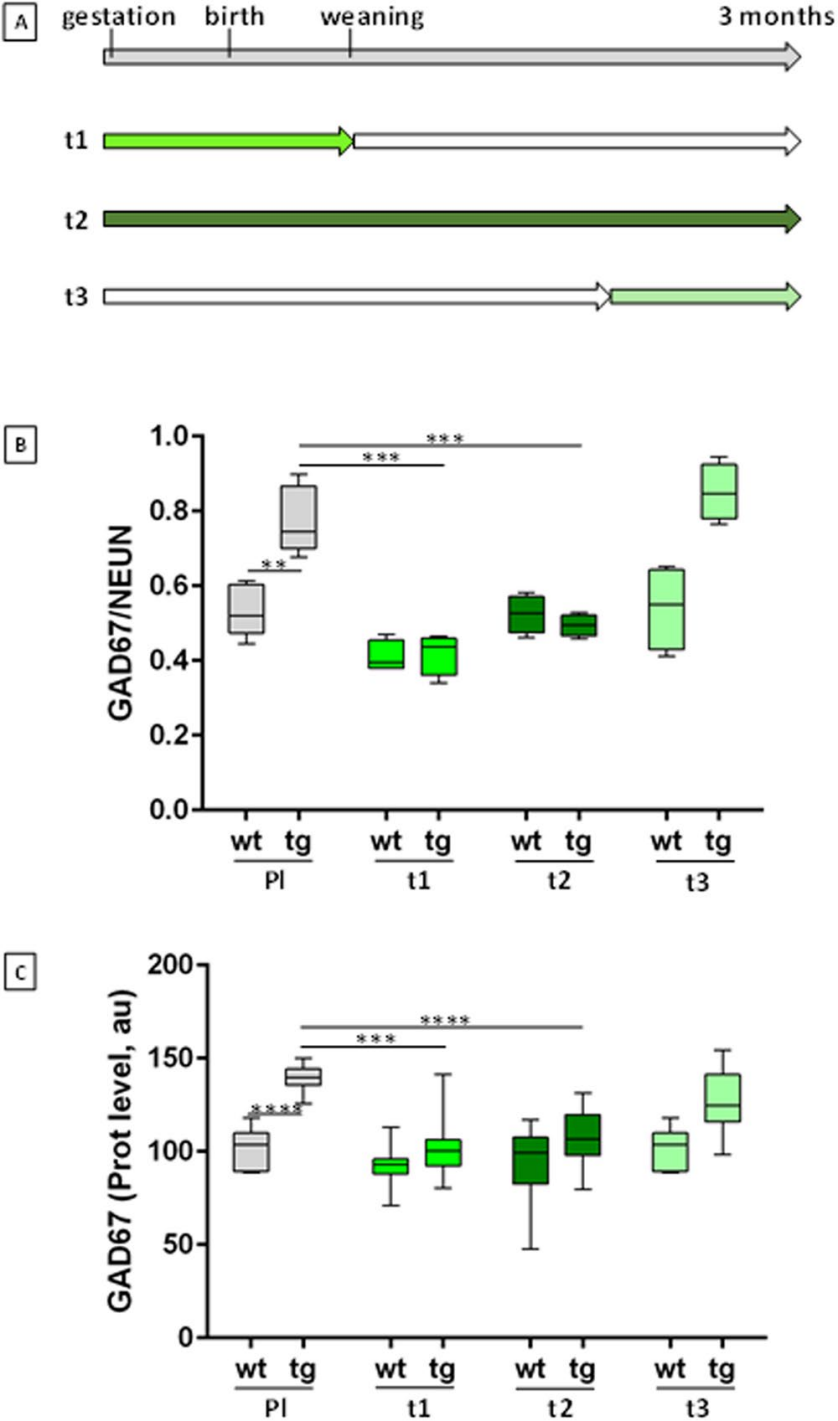
EGCG treatment during brain development of mBACtgDyrk1a mice. (**A**) Timelines of treatment of mBACtgDyrk1a transgenic (tg) mice from gestation through adulthood with normal food pellets or pellets (SAFE company) containing 600 mg/kg MGTE (decaffeinated Lifeextension extract containing 45% EGCG) and corresponding to a daily dose of 50 mg/kg EGCG for a 25 g mouse. Food consumption was similar for wt and tg animals. T1 treatment started at gestation and continued through weaning. T2 treatment started at gestation and continued until 90 days. T3 treatment started at P60 and continued until P90. Grey indicates standard food (placebo); green indicates treatment on each timeline. (**B**) Quantification of GAD67+ neuron fraction of NeuN+ neurons in immunohistochemically stained sections of stratum radiatum of control (n = 5) or treated (n = 4) wildtype (WT) and transgenic (TG) mice: serial sagittal brain cryosections (50 µm) were cut on a cryostat and immunohistochemistry was performed with GAD67 (Millipore MAB5406) and NeuN (Millipore ABN 78) antibodies. NeuN-positive and GAD67-positive neuron densities were assessed with StereoInvestigator (MBF) in stratum radiatum in parasagittal slices (+0.36 mm). n = 5 for wt and tg; n = 4 for treated wt and treated tg. (**C**) Relative GAD67 levels in hippocampus of control or treated WT and TG mice. Arbitrary units (au) of GAD67 levels were normalised to total protein levels and to controls (n = 10). Two ways ANOVA were performed followed by an Holm–Sidak multiple comparison procedure with ***p < 0.001, ****p < 0.0001.

mBACtgDyrk1a is a monogenic model expressing three copies of *Dyrk1a* that exhibits developmental and cognitive defects^7^. We first performed a set of experiments on mBACtgDyrk1a and wildtype mice from the same litters. Treatment T1 was given prenatally to gestating mothers and stopped at weaning; treatment T2 was given prenatally to gestating mothers and continued through adulthood; and treatment T3 was given to 2–3-month-old adult mice for one month (Fig. 1A).

Brains of treated animals were collected at 3 months, and sagittal slices were analysed for marker density with stereological techniques. Stratum radiatum of untreated transgenic mice showed increased density of GAD67 neurons in comparison with wildtype animals (*p* < 0.01) (Fig. 1B and Table 1). Treating adult mice with EGCG (T3) did not affect this finding. However, prenatal EGCG treatment corrected the increase (T1 and T2), and this correction persisted after cessation of treatment at weaning (T1). Two way ANOVA revealed a significant effect of genotype and treatment (genotype: p = 0.0018; treatment p < 0.0001) and a significant interaction between genotype and treatment (p = 0.0031). A Holm–Sidak multiple comparison procedure revealed that GAD67 relative density is significantly rescued both after T1 treatment (p_tg_ = 0.0002) or after T2 treatment (p_tg_ = 0.0005). After treatment T3 differences were not significant. These results support the hypothesis that overaccumulation of GABAergic neurons in this model takes place during early development.

**Table 1:** Treatments effects in mBACtgDyrk1a hippocampus.

Density		T1		T2		T3	
		F	P value	F	P value	F	P value
GAD67 neurons density/Neun	Interaction	F (1, 14) = 12,76	P = 0,0031	F (1, 14) = 18,74	P = 0,0007	F (1, 14) = 0,6947	P = 0,4186
	Genotype	F (1, 14) = 14,85	P = 0,0018	F (1, 14) = 11,31	P = 0,0046	F (1, 14) = 44,62	P < 0,0001
	Treatment	F (1, 14) = 55,17	P < 0,0001	F (1, 14) = 21,88	P = 0,0004	F (1, 14) = 0,9450	P = 0,3475
**Protein levels**							
GAD67	Interaction	F (1, 36) = 9,916	P = 0,0033	F (1, 40) = 7,254	P = 0,0103	F (1, 35) = 2,316	P = 0,1370
	Genotype	F (1, 36) = 33,88	P < 0,0001	F (1, 40) = 34,27	P < 0,0001	F (1, 35) = 56,71	P < 0,0001
	Treatment	F (1, 36) = 28,40	P < 0,0001	F (1, 40) = 19,45	P < 0,0001	F (1, 35) = 2,316	P = 0,1370
GAD65		F (1, 39) = 6,545	P = 0,0145	F (1, 42) = 0,1534	P = 0,6973	F (1, 36) = 0,2613	P = 0,6123
		F (1, 39) = 13,71	P = 0,0007	F (1, 42) = 31,62	P < 0,0001	F (1, 36) = 39,19	P < 0,0001
		F (1, 39) = 6,511	P = 0,0148	F (1, 42) = 1,082	P = 0,3041	F (1, 36) = 0,2301	P = 0,6344
VGAT1		F (1, 37) = 3,352	P = 0,0752	F (1, 38) = 1,726	P = 0,1968	F (1, 35) = 5,556	P = 0,0241
		F (1, 37) = 26,76	P < 0,0001	F (1, 38) = 37,29	P < 0,0001	F (1, 35) = 8,755	P = 0,0055
		F (1, 37) = 2,844	P = 0,1002	F (1, 38) = 8,845	P = 0,0051	F (1, 35) = 5,364	P = 0,0265
PSD95		F (1, 38) = 35,74	P < 0,0001	F (1, 43) = 15,17	P = 0,0003		
		F (1, 38) = 31,22	P < 0,0001	F (1, 43) = 32,35	P < 0,0001		
		F (1, 38) = 60,35	P < 0,0001	F (1, 43) = 6,183	P = 0,0169		
GLUR2		F (1, 40) = 0,7223	P = 0,4005	F (1, 44) = 2,214	P = 0,1439	F (1, 37) = 6,726	P = = 0,0135
		F (1, 40) = 13,28	P = 0,0008	F (1, 44) = 9,983	P = 0,0029	F (1, 37) = 8,465	P = 0,0061
		F (1, 40) = 16,26	P = 0,0002	F (1, 44) = 36,55	P < 0,0001	F (1, 37) = 7,370	P = 0,0100
NR2A		F (1, 39) = 3,725	P = 0,0609	F (1, 42) = 8,178	P = 0,0066	F (1, 36) = 20,05	P < 0,0001
		F (1, 39) = 11,09	P = 0,0019	F (1, 42) = 4,991	P = 0,0309	F (1, 36) = 0,0003	P = 0,9859
		F (1, 39) = 0,0944	P = 0,7602	F (1, 42) = 0,1399	P = 0,7103	F (1, 36) = 18,57	P = 0,0001
VGLUT1		F (1, 38) = 21,63	P < 0,0001	F (1, 42) = 0,2429	P = 0,6247	F (1, 35) = 4,167	P = 0,0488
		F (1, 38) = 38,92	P < 0,0001	F (1, 42) = 4,343	P = 0,0433	F (1, 35) = 0,02035	P = 0,8874
		F (1, 38) = 30,27	P < 0,0001	F (1, 42) = 3,706	P = 0,0610	F (1, 35) = 4,908	P = 0,0333
VGAT1/VGLUT1		F (1, 38) = 25,91	P < 0,0001	F (1, 42) = 5,230	P = 0,0273	F (1, 33) = 0,9883	P = 0,3274
		F (1, 38) = 1,337	P = 0,2549	F (1, 42) = 14,00	P = 0,0005	F (1, 33) = 7,328	P = 0,0107
		F (1, 38) = 30,40	P < 0,0001	F (1, 42) = 19,61	P < 0,0001	F (1, 33) = 0,2727	P = 0,6050

Quantification of synaptic markers was performed on the second half of brains from the same mice. Transgenic mice overexpressed GAD67 by 1.4-times the levels of wildtype mice (*p* < 0.0001) (Fig. 1C). T1 treatment was sufficient to correct GAD67 overexpression in the hippocampus as revealed by t-test analyses of the protein levels: comparison of wild type (wt) and transgenic, of wt and transgenic after treatment, and of transgenic and transgenic after treatments T1, T2 and T3 (Sup. Table 1). This correction persisted when treatment continued into adulthood (T2; 93% correction), but was weaker when treatment was delayed until adulthood (T3; 24% correction) (Fig. 1C). A two way ANOVA revealed a significant effect of genotype, treatment and interaction for GAD67 level by T1 and T2 treatments. With T3 we observed only a genotype effect (Table 1). A Holm–Sidak multiple comparison procedure revealed that GAD67 protein level is significantly corrected by treatment T1(p_tg_ = 0.0001) and by treatment T2 (p_tg_ = 2.6 × 10^−5^). Similar results were found for GAD65 with T1, for GABA transporter and for PSD95 (DLG4) with T1 and T2. For VGAT1 treatment effect was significant for T1 (p_tg_ = 0.03) with higher significancy for T2 (p_tg_ = 0.0007) and T3. Prenatal treatment is necessary for a strong correction of gabaergic markers. In addition, we observed that transgenic mice had decreased levels of synaptic markers, GLUR2 and NR2A, from the glutamate pathway:treatment T1,T2 and T3 corrected GLUR2 alterations and T2, T3 corrected NR2A alterations. Corrections of excitatory markers were more pronounced after T2 or T3 treatment. A significant treatment effect (T1 and T2) was observed for the ratio VGAT1/VGLUT1 with T1 and T2 (p < 0.0001) Table 1. The same analysis was performed on cortex proteins: treatment T1 induces a significant correction of the level of GABA markers: GAD67, GAD65, VGAT1; a treatment effect was also visible for NR1, NR2A, GLUR2 and VGLUT1; a significant treatment effect was observed for the ratio VGAT1/VGLUT1 with the three treatment protocols (Table 2 and Sup. Table 2).

**Table 2:** Treatments effects in mBACtgDyrk1a cortex.

Protein levels		T1		T2		T3	
		F	P value	F	P value	F	P value
GAD67	Interaction	F (1, 37) = 3,069	P = 0,0881	F (1, 35) = 1,973	P = 0,1689	F (1, 36) = 0,1422	P = 0,7083
	Genotype	F (1, 37) = 46,31	P < 0,0001	F (1, 35) = 43,48	P < 0,0001	F (1, 36) = 57,50	P < 0,0001
	Treatment	F (1, 37) = 9,028	P = 0,0048	F (1, 35) = 0,4603	P = 0,5020	F (1, 36) = 0,2822	P = 0,5985
GAD65		F (1, 40) = 3,202	P = 0,0811	F (1, 41) = 1,956	P = 0,1694	F (1, 37) = 4,310	P = 0,0449
		F (1, 40) = 35,79	P < 0,0001	F (1, 41) = 32,78	P < 0,0001	F (1, 37) = 30,60	P < 0,0001
		F (1, 40) = 6,152	P = 0,0174	F (1, 41) = 0,3709	P = 0,5459	F (1, 37) = 5,555	P = 0,0238
VGAT1		F (1, 41) = 1,371	P = 0,2489	F (1, 42) = 3,772	P = 0,0594	F (1, 38) = 19,26	P < 0,0001
		F (1, 41) = 46,83	P < 0,0001	F (1, 42) = 25,28	P < 0,0001	F (1, 38) = 14,04	P = 0,0006
		F (1, 41) = 23,41	P < 0,0001	F (1, 42) = 2,822	P = 0,1010	F (1, 38) = 22,89	P < 0,0001
GLUR2		F (1, 40) = 0,8901	P = 0,3511	F (1, 41) = 0,1540	P = 0,6967	F (1, 37) = 0,4270	P = = 0,5175
		F (1, 40) = 0,1568	P = 0,6942	F (1, 41) = 1,520	P = 0,2247	F (1, 37) = 1,577	P = 0,2170
		F (1, 40) = 1,852	P = 0,1812	F (1, 41) = 2,040	P = 0,1608	F (1, 37) = 0,5287	P = 0,4717
NR1		F (1, 39) = 5,218e-006	P = 0,9982	F (1, 41) = 0,6596	P = 0,4214	F (1, 37) = 0,07995	P = 0,7789
		F (1, 39) = 0,9666	P = 0,3316	F (1, 41) = 6,369	P = 0,0156	F (1, 37) = 1,748	P = 0,1942
		F (1, 39) = 6,660	P = 0,0137	F (1, 41) = 10,32	P = 0,0026	F (1, 37) = 0,1150	P = 0,7365
NR2A		F (1, 41) = 3,851	P = 0,0565	F (1, 42) = 7,293	P = 0,0099	F (1, 37) = 2,626	P = 0,1136
		F (1, 41) = 0,1100	P = 0,7418	F (1, 42) = 0,2296	P = 0,6343	F (1, 37) = 2,558	P = 0,1183
		F (1, 41) = 6,238	P = 0,0166	F (1, 42) = 2,058	P = 0,1588	F (1, 37) = 0,7885	P = 0,3803
VGLUT1		F (1, 41) = 3,851	P = 0,0565	F (1, 42) = 8,4074	P = 0,0059	F (1, 38) = 2,099	P = 0,1556
		F (1, 41) = 0,1100	P = 0,7418	F (1, 42) = 0,6196	P = 0,9803	F (1, 38) = 3,150	P = 0,0839
		F (1, 41) = 6,238	P = 0,0166	F (1, 42) = 3,0801	P = 0,0866	F (1, 38) = 1,129	P = 0,2946
VGAT1/VGLUT1		F (1, 41) = 34,80	P < 0,0001	F (1, 42) = 23,99	P < 0,0001	F (1, 38) = 8,279	P = 0,0065
		F (1, 41) = 8,857	P = 0,0048	F (1, 42) = 19,05	P < 0,0001	F (1, 38) = 21,85	P < 0,0001
		F (1, 41) = 30,99	P < 0,0001	F (1, 42) = 26,03	P < 0,0001	F (1, 38) = 9,994	P = 0,0030

### Prenatal EGCG treatment corrects GAD67 levels in Dp(16)1Yey mice

**Figure 2:**
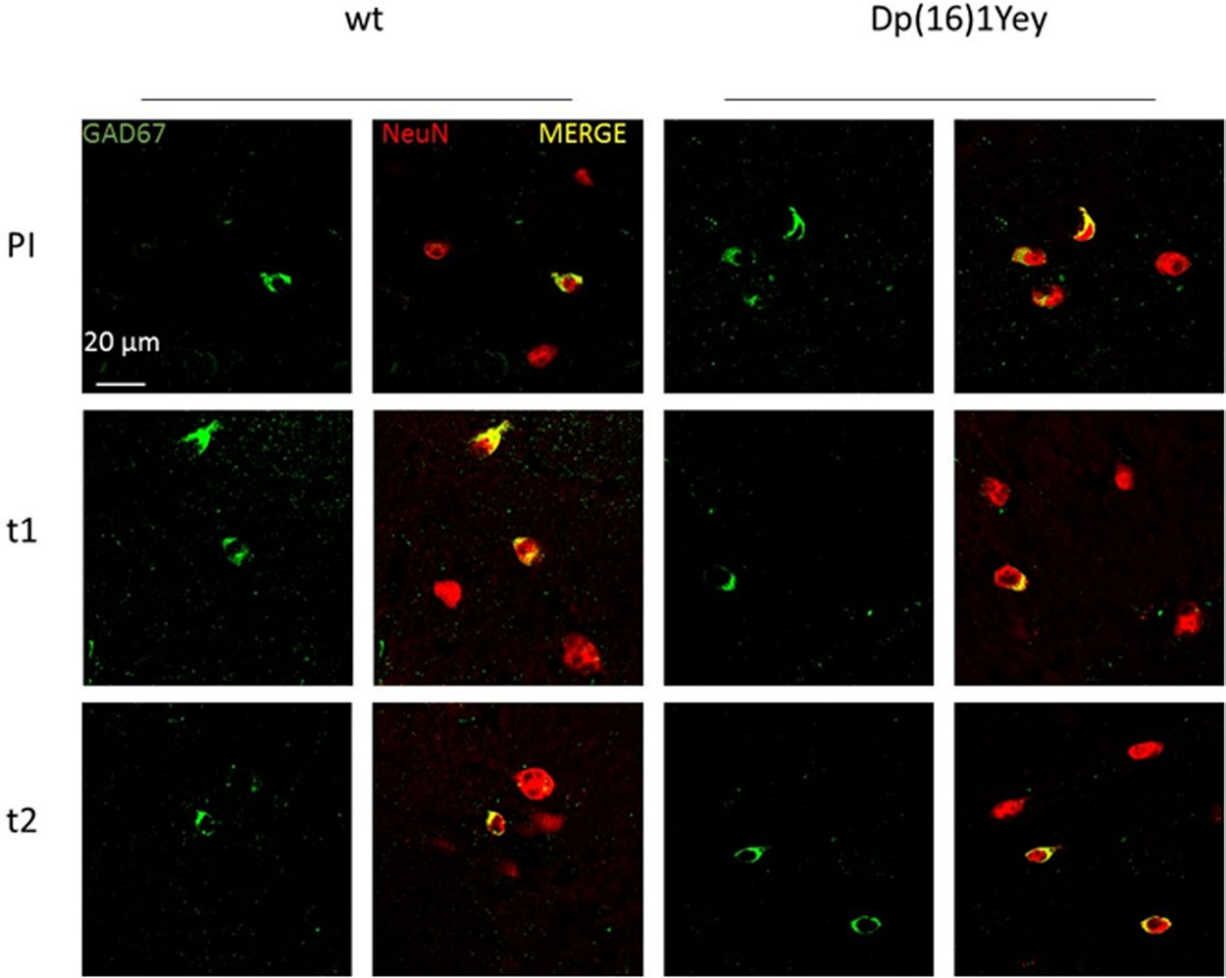
Representative images of immunohistochemical staining of GAD67 (green) and NeuN (red) in stratum radiatum of wild type (WT) and trisomic Dp(16)1Yey mice given placebo (Pl), T1 or T2 treatment.

**Figure 3:**
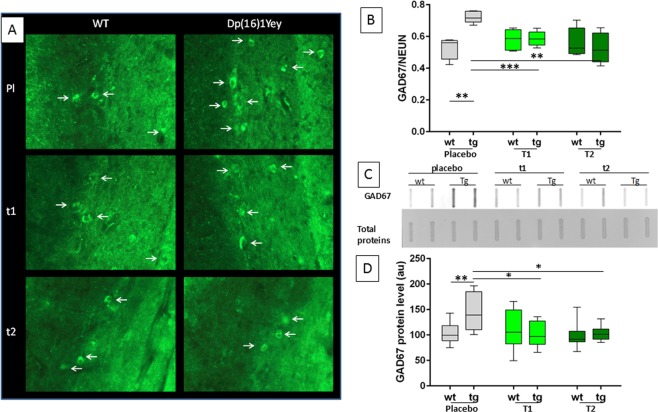
Effects of EGCG treatment on GAD67 neurons during development of Dp16(1)Yey mice. (**A**) Representative fluorescent micrographs of immunohistochemically stained GAD67+ neurons in stratum radiatum of wildtype (WT) and Dp16(1)Yey trisomic (TS) adult mice after placebo (Pl), T1, or T2 treatment. White arrows indicate labelled neurons. (**B**) Quantification of GAD67+ neuron fraction of NeuN+ neurons in sections of stratum radiatum of WT and Dp16(1)Yey adult mice (n = 5). (**C**) Representative image of slot blots used for protein level quantification: top, GAD67 antibody, bottom, Ponceau staining. (**D**) Relative GAD67 levels in hippocampus of control or treated WT and TS mice. Two ways ANOVA were performed followed by an Holm–Sidak multiple comparison procedure with *p < 0.05, **p < 0.01.

Then, we analysed effects of T1 and T2 treatment in the more genetically complex Dp(16)1Yey model, in which we previously observed that GAD67 neuron density is increased in stratum radiatum similarly to mBACtgDyrk1a mice^7^. We applied the same protocol as used for mBACtgDyrk1a experiments to mice divided in four groups: placebo-fed wildtype, placebo-fed Dp(16)1Yey trisomic, EGCG-fed wildtype, and EGCG-fed trisomic mice. Colocalisations of GAD67 and Neun signals were observed in confocal images of stratum radiatum of placebo treated and T1, T2 treated mice (Fig. 2). Sagittal slices were analysed for marker density with stereological techniques. T1-treated mice displayed a correction of increased GAD67 neuron density in stratum radiatum: a significant interaction was observed using a two way ANOVA (Table 2) (Fig. 3A,B); a Holm–Sidak multiple comparison procedure revealed a significant correction of the ratio GAD67/Neun after T1 treatment (p_ts_ = 0.0008) and T2 treatment (p_ts_ = 0.0027). GAD67 expression in hippocampus (Fig. 3C) is significantly increased in Dp(16)1Yey (*p* = 0.0004). Expression data were analysed by t-tests (Sup. Tables 3 and 4) and by two ways ANOVA (Table 3) and revealed a significant interaction and a significant treatment effect for T1 and T2 treatment. A Holm–Sidak multiple comparison procedure revealed a correction of the GAD67 protein level after T1 treatment close to significancy (p_ts_ = 0.1) and a significant correction for T2 treatment (p_ts_ = 0.03). A treatment effect was also observed for GAD65 after T1 and T2 and for VGAT1 (for T2 p_ts_ = 0.05). A treatment effect was observed for GLUR2 after T1 (p_ts_ = 0.03). (and after T2 (p_tg_ = 6 × 10^−4^). Although there is no genotype effect for VGLUT1 there is a treatment effect after T2 (Table 3 and Sup. Table 3). A very significant treatment effect was observed for the ratio VGAT1/VGLUT1 with T1 and T2 (Table 3). In cortex treatment T2 induced a significant correction of GAD67and GAD65. A significant correction was also observed for VGAT1/VGLUT1 after T2. (Table 4 and Sup. Table 4). Because similar effects were observed in the single gene model (mBACtgDyrk1a) and partial trisomy model (Dp(16)1Yey), we propose that DYRK1A overexpression is the main cause of GABAergic over accumulation in DS models and of alteration of the ratio VGAT1/VGLUT1.

**Table 3:** Treatments effects in Dp(16)1Yey hippocampus.

Density		T1		T2	
		F	P value	F	P value
GAD67 neurons density/Neun	Interaction	F (1, 15) = 13,05	P = 0,0026	F (1, 15) = 10,20	P = 0,0061
	genotype	F (1, 15) = 14,71	P = 0,0016	F (1, 15) = 4,737	P = 0,0459
	Treatment	F (1, 15) = 2,906	P = 0,1088	F (1, 15) = 5,197	P = 0,0377
**Protein levels**					
GAD67	Interaction	F (1, 36) = 6,463	P = 0,0155	F (1, 39) = 5,009	P = 0,0310
	genotype	F (1, 36) = 2,524	P = 0,1209	F (1, 39) = 8,170	P = 0,0068
	Treatment	F (1, 36) = 3,264	P = 0,0792	F (1, 39) = 8,384	P = 0,0062
GAD65	Interaction	F (1, 36) = 5,339	P = 0,0267	F (1, 36) = 6,773	P = 0,0134
	genotype	F (1, 36) = 1,336	P = 0,2554	F (1, 36) = 2,270	P = 0,1406
	Treatment	F (1, 36) = 3,707	P = 0,0621	F (1, 36) = 6,133	P = 0,0181
VGAT1	Interaction	F (1, 35) = 0,6496	P = 0,4257	F (1, 38) = 6,439	P = 0,0154
	genotype	F (1, 35) = 16,96	P = 0,0002	F (1, 38) = 11,55	P = 0,0016
	Treatment	F (1, 35) = 11,48	P = 0,0018	F (1, 38) = 17,73	P = 0,0002
GLUR2	Interaction	F (1, 38) = 2,348	P = 0,1338	F (1, 38) = 9,081	P = 0,0046
	genotype	F (1, 38) = 0,8522	P = 0,3618	F (1, 38) = 0,2135	P = 0,6467
	Treatment	F (1, 38) = 4,214	P = 0,0470	F (1, 38) = 4,355	P = 0,0437
VGLUT1	Interaction	F (1, 33) = 5,490	P = 0,0253	F (1, 34) = 10,12	P = 0,0031
	genotype	F (1, 33) = 0,4361	P = 0,5136	F (1, 34) = 2,353	P = 0,1343
	Treatment	F (1, 33) = 0,2981	P = 0,5888	F (1, 34) = 24,94	P < 0,0001
VGAT1/VGLUT	Interaction	F (1, 33) = 31,04	P < 0,0001	F (1, 33) = 42,19	P < 0,0001
	genotype	F (1, 33) = 27,94	P < 0,0001	F (1, 33) = 33,47	P < 0,0001
	Treatment	F (1, 33) = 34,99	P < 0,0001	F (1, 33) = 4,400	P = 0,0437

**Table 4:** Treatments effects in Dp(16)1Yey cortex.

		T1		T2	
**Protein levels**					
GAD67	Interaction	F (1, 39) = 3,664	P = 0,0629	F (1, 39) = 1,639	P = 0,2080
	genotype	F (1, 39) = 1,215	P = 0,2770	F (1, 39) = 4,317	P = 0,0444
	Treatment	F (1, 39) = 2,334	P = 0,1346	F (1, 39) = 12,70	P = 0,0010
GAD65	Interaction	F (1, 36) = 7,208	P = 0,0109	F (1, 39) = 2,314	P = 0,1363
	genotype	F (1, 36) = 1,156	P = 0,2894	F (1, 39) = 7,347	P = 0,0099
	Treatment	F (1, 36) = 0,1360	P = 0,7145	F (1, 39) = 5,090	P = 0,0297
VGAT1	Interaction	F (1, 39) = 0,6701	P = 0,4180	F (1, 42) = 1,942	P = 0,1708
	genotype	F (1, 39) = 14,73	P = 0,0004	F (1, 42) = 30,24	P < 0,0001
	Treatment	F (1, 39) = 0,9720	P = 0,3302	F (1, 42) = 3,062	P = 0,0874
GLUR2	Interaction	F (1, 38) = 2,145	P = 0,1512	F (1, 39) = 1,447	P = 0,2362
	genotype	F (1, 38) = 1,179	P = 0,2845	F (1, 39) = 3,995	P = 0,0526
	Treatment	F (1, 38) = 7,807	P = 0,0081	F (1, 39) = 49,44	P < 0,0001
VGLUT1	Interaction	F (1, 34) = 0,2892	P = 0,5942	F (1, 36) = 1,109	P = 0,2993
	genotype	F (1, 34) = 2,742	P = 0,1070	F (1, 36) = 6,225	P = 0,0173
	Treatment	F (1, 34) = 1,710	P = 0,1998	F (1, 36) = 19,12	P = 0,0001
VGAT1/VGLUT	Interaction	F (1, 32) = 0,8482	P = 0,3639	F (1, 36) = 14,69	P = 0,0005
	genotype	F (1, 32) = 19,02	P = 0,0001	F (1, 36) = 60,98	P < 0,0001
	Treatment	F (1, 32) = 1,683	P = 0,2038	F (1, 36) = 38,93	P < 0,0001

### Prenatal EGCG treatment restores NOR memory

**Figure 4:**
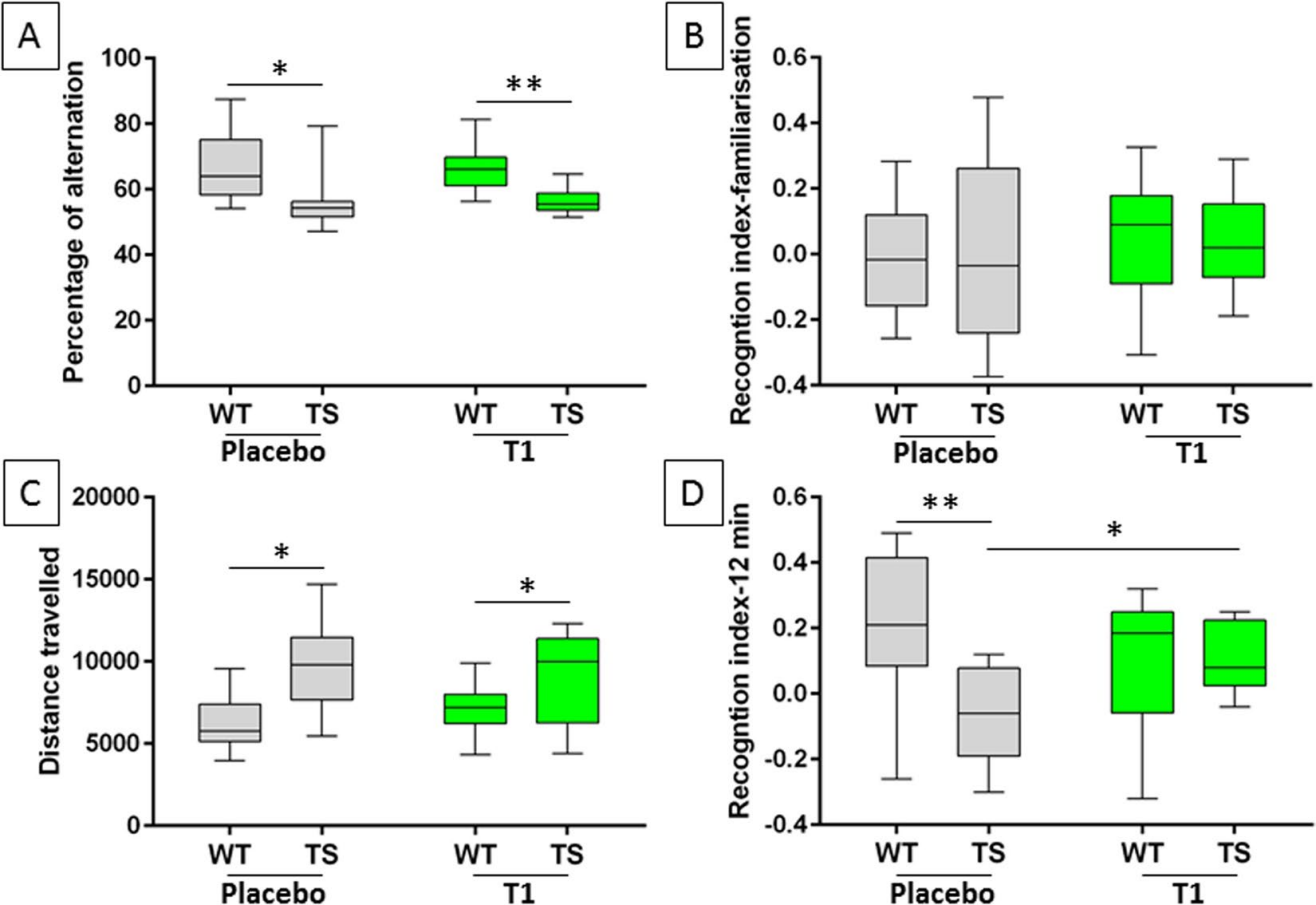
Effects of EGCG treatment on Y maze and novel object recognition memory of Dp16(1)Yey mice. Y maze tests and Novel object recognition memory tests were performed on control wildtype (WT) (n = 14) and Dp16(1)Yey transgenic (TG) (n = 11) and treated WT (n = 16) and TG (n = 8) mice at the end of T1 treatment (P90). (**A**) Alternation performance in a Y-maze test. (**B**) Recognition index between familiarisation phase. (**C**) Quantification of videotracked distance mice travelled on the first day during habituation to the arena for 30 min. (**D**) Recognition index for mice exploring novel and familiar objects during the second day of novel object recognition memory tests. Recognition index (RI) was defined as RI = (exploration time_novel objects_ − exploration time_familiar_ objects)/(exploration time_novel objects_ + exploration time_familiar_ objects). Analysis of exploration time was performed during 12 min; Two ways ANOVA were performed followed by an Holm–Sidak multiple comparison procedure with *p < 0.05.

Dp(16)1Yey mice have been shown to be impaired for the Y maze and the novel object tests. Y Maze Spontaneous Alternation is a behavioral test for measuring the willingness of rodents to explore new environments. We observed a deficit in percent of alternation for Dp(16)1Yey mice compared to wildtype. However T1 treatment did not induce any rescue (Fig. 4A).

The novel object recognition test, evaluated by differences in exploration times of novel and familiar objects, is based on the innate tendency of rodents to differentially explore novel objects over familiar ones^26^. In rodents, the test is sensitive to direct or chemical lesions in the hippocampus^27,28^. In the medial temporal lobe, the hippocampus and adjacent cortical areas, including entorhinal, perirhinal, and parahippocampal cortexes, are involved in normal memory function. The hippocampus is responsible for long-term object recognition^29^.

During the familiarisation phase recognition index is not significantly different in Dp(16)1Yey and there is no treatment effect on this phase (Fig. 4B). During the first sessions of object recognition tests in which mice are habituated to the testing arena, activity videotracking, analysed by two ways ANOVA, indicated hyperactivity of placebo-treated Dp(16)1Yey mice (genotype effect p < 0.0001) (Fig. 4C). Treatment had no significant effect on hyperactivity. Object recognition memory performance was then specifically evaluated after a retention phase of 24 h by analysing the time mice spent exploring familiar versus novel objects. Euploid mice, regardless of EGCG treatment, were able to discriminate between novel and familiar objects, indicating normal recognition memory during the 12-min recording time (Fig. 4D). In contrast, vehicle-treated Dp(16)1Yey mice did not show any significant exploratory preference towards novel objects. However, T1-treated Dp(16)1Yey mice clearly differentiated between two objects: two ways ANOVA revealed significant genotype and treatment effects and a significant interaction; a Holm–Sidak multiple comparison procedure showed a significant effect of treatment on Dp(16)1Yey mice (p = 0.01) indicating that EGCG treatment was able to restore novel object recognition memory more than two months after cessation of treatment.

## Discussion

Using stereological measurements we have previously shown alterations of gabaergic interneurons by assessing GAD67+ neurons density in DS mouse models. We documented these variations in stratum radiatum of mBACtgDyrk1a, hYACtgDyrk1a and Dp(16)1Yey^7^; here we compared ratios of GAD67+ cells/Neun+ cells: we found that increased dosage of DYRK1A is associated with an increase of 30% of this ratio in mBACtgDyrk1a stratum radiatum; in Dp(16)1Yey we observed an increase of 30%. These alterations are not limited to stratum radiatum: stereological experiments performed on colliculus, a region rich in interneurons, revealed an increase of 43% of the ratio GAD67+ cells/Neun+ cells in Dp(16)1Yey (Fig. 2 Supplementary Data).

These observations were in agreement with most of the previously published studies: Ts65Dn mice display an increased number of GABAergic interneurons in the cortex and hippocampus: parvalbumin positive cells are increased in CA1 of Ts65Dn mice at P15^30^; however this observation is not reproduced in a study performed with P15 Dp(16)1Yey mice^31^; in adult Ts65dn an increase in the number of inhibitory neurons in CA1 and CA3, mainly interneurons expressing calbindin, calretinin, NPY and VIP, was reported whereas parvalbumin cell numbers were not affected^32,33^; in adult Dp(16)1Yey the number of NPY+ inhibitory interneurons was reported increased although the density of parvalbumin+ cells was not significantly increased in stratum pyramidale, radiatum and lacunosum moleculare. Alterations of inhibitory neuropil in hippocampus have also been reported and they include a decrease of excitatory boutons (VGLUT1+) and an increase of inhibitory boutons (VGAT1+)^32,34,35^.

We have also shown previously that protein levels of inhibitory and excitatory markers are altered in hippocampus, cortex and cerebellum of mBACtgDyrk1a, Ts65Dn and Dp(16)1Yey: here we observed an increase of levels of GAD67, GAD65 and VGAT1 and a decrease of levels of GLUR1, GLUR2, NR1 and NR2A in hippocampus of mBACtgDyrk1a as compared to wild type animals; in hippocampus of Dp(16)1Yey mice we observed an increase of levels of GAD67, GAD65, VGAT1 and a decrease of levels of GLUR1, NR1 and NR2A. Interestingly in mice with one active copy of Dyrk1a gene (Dyrk1a+/−) we previously observed a decreased level of GAD67 and PSD95 and an increased level of GLUR1, GLUR2, NR1 and NR2A^7^. These results suggest a link between DYRK1A dose and level of gabaergic and glutamatergic markers.

In humans few studies have been performed on brain samples from patients with Down syndrome: the first studies were performed on aged individuals: Golgi studies revealed a poverty of granular cells in three Brodman areas of two DS cases^36^; in another study the number of calbindin+ and parvalbumin+ neurons was found reduced in elderly persons with DS^37^. However brains of these patients presented histopathological features of Alzheimer’s disease, a pathology which has been associated with gabaergic dysfunction. On contrary in hippocampus of DS embryos density of calretinin positive cells is increased^5^, suggesting that further quantitative studies should be performed on brains from young individuals.

In mice morphological and cellular data seem to suggest that there is an increased density of inhibitory interneurons in stratum radiatum and an increased level of GAD67, GAD65 and VGAT1 protein. However the functional consequences of these alterations are not clearly established: hippocampal long-term potentiation is suppressed in Ts65Dn CA1 and rescued after suppressing inhibition with picrotoxin, a GABA(A) receptor antagonist^6^; a similar observation was made for theta burst induced LTP^38^; however LTP was found normal in CA3 recordings of synaptically connected neurons^39^. Genetic correction of Dyrk1a gene copy number in Ts65Dn rescues the density of GABAergic and glutamatergic synapse markers in the molecular layer of the hippocampus and CA1 hippocampal LTP^40^; however other electrophysiological studies suggest that gabaergic signalling could be depolarizing rather than hyperpolarizing in adult animals^41^. Using primary cultures and acute hippocampal slices it was shown that developmental excitatory-to-inhibitory GABA polarity switch is delayed by 2 days in young Ts65Dn mice^42^ and that these changes lead to a delay in maturation of nascent neural circuits.

In cortex we have shown in mBACtgDyrk1A and Dp(16)1Yey mice an increased level of Gad67 level suggesting that regulatory consequences of an increased level of DYRK1A are the same as in hippocampus; the ratio of Gad67+ neurons/Neun+ neurons was also found increased in colliculus of Dp(16)1Yey mice, reminding our observation in hippocampus; however in this brain region density of Gad67+ neurons is not altered but density of Neun+ neurons is decreased. In layer 4 of somatosensory cortex of Ts65Dn mice spontaneous synaptic inputs are decreased in frequency but the balance between excitatory and inhibitory drive appears similar between the two genotypes^43^; in cerebellum other data suggest that tonic gabaergic inhibition could be less efficient in Ts65dn^44^. These results suggest that developmental impact of trisomy is depending on the brain regions.

In various experiments targeting DYRK1A, EGCG or green tea extracts have been compared to harmine, a more potent inhibitor of DYRK1A but which is also inducing tremors. We have used an EGCG-enriched green tea extract as previous studies have shown that EGCG can cross the blood brain barrier^25,45^ and the placental barrier^46^. Given prenatally this extract (MGTE) was shown to rescue the ratio 4R-tau/3Rtau, controlled by DYRK1A, in Ts65Dn mouse brains^47^.

Our results reveal that treatment targeting DYRK1A is sufficient to correct GABAergic pathway alterations when given prenatally to single gene (mBACtgDYRK1A) or trisomic [Dp16(1)Yey] mouse models. In mBACtgDYRK1A model we observed these corrections in hippocampus and cortex. These corrections are still present 69 days after cessation of treatment. Reducing inhibition levels with various pharmacological approaches has been shown to promote recovery from cognitive impairment in Ts65Dn mice^6,48,49^.

A link has been shown also between GABA plasticity and cognition in Ts65Dn treated chronically with fluoxetine: treatment normalizes GABA release by synaptosomes from hippocampus and spatial memory^50^. Involvement of GABAergic neurotransmission into alterations in novel place and novel object recognition was also shown by treating Ts65Dn mice with a GABAB receptor antagonist^51^. Rescuing effects of EGCG treatments on cognition paradigms have been previously shown on TgDyrk1a and Ts65Dn. Here a prenatal treatment does not correct Y maze deficit, a test known to be impaired by alterations of NMDA levels^52^. This treatment allows to correct recognition index in a novel object recognition paradigm: T1 and T2 corrects GABAergic markers and the effect of T2 is stronger on glutamatergic markers. These results suggest correction of glutamatergic pathways is necessary for correcting Y maze alterations; on the contrary correction of gabaergic pathways, which is maintained after cessation of treatment (T1), is sufficient to rescue deficits observed with novel object recognition paradigm.

Comparison with a previous report of postnatal EGCG treatment showing no long-term effect of EGCG treatment (subcutaneaous injection of 25 mg/kg at P3–P15)^21^ suggests that prenatal action is necessary for long-term effects on GABA pathway. In mice, GABAergic neurons arise in the medial and caudal ganglionic eminences in ventral telencephalon and migrate first tangentially from their progenitor niche to the proper cortical region and then radially through the cortical plate to reach their final laminar location^53–55^. Accordingly, EGCG likely acts during the early phases in which DYRK1A expression is important to determine inhibitory neuron destiny. Interestingly we also show that this prenatal treatment is able to rescue level of PSD95, a major regulator of synaptic maturation, disruption of which is observed in schizophrenia and autism^56^.

We have shown previously that EGCG treatment, given at adult stage, rescues latency and thigmotaxy of 4–5 months old Ts65dn mice performing a Morris water maze test^17^. A similar experiment with purified EGCG was performed on younger adolescent Ts65Dn mice and could not reproduce either the genotype effect or the treatment effect.^57^ Here we show that prenatal treatment of Dp(16)1Yey mice when prolonged until adulthood induces a correction both of synaptic markers associated with GABAergic pathways and of markers associated with glutamatergic pathways. To get the most efficient rescue it seems therefore necessary to combine actions targeting these two pathways either with a prenatal EGCG treatment (targeting GABA pathway) and a continued EGCG treatment (targeting glutamate pathway) till adult stage or by combining prenatal EGCG treatment with a different pharmacological approach targeting glutamatergic pathway. As previously proposed, *Dyrk1a* is not the only overexpressed gene that may cause these phenotypes. Role of overexpression of GIRK2 has been hypothesized to explain GABAB/GABAA ratios evoked by stimulation of stratum lacunosum moleculare^58^. Olig1 and olig2 duplication have been linked to increased density of parvalbumin positive cells in CA1 of Ts65Dn^30^. Beneficial effect of increased aerobic exercise points toward an involvement of a BDNF related pathway^59^. Nevertheless, the cellular, biochemical, and functional rescues induced by prenatal treatment targeting DYRK1A are promising. Recently, administration of ALGERNON, a potent DYRK1A inhibitor (IC50 = 76.9 nM), to pregnant dams rescued aberrant cortical formation in DS mouse embryos (Ts1Cje) and prevented the development of abnormal behaviors in DS offspring^60^. However it has been recently proposed that heterozygous disruption of DYRK1A causes a distinctive clinical syndrome, MRD7, that is characterized by the presence of mild to severe ID, microcephaly, intrauterine growth retardation, facial dimorphisms, impaired motor functions and behavioural problems associated to one form of autism^61^. In prenatal therapy with ALGERNON, treated WT offspring showed trend of impaired learning behaviors suggesting that prenatal inhibition of DYRK1A should be finely tuned to avoid deleterious effects. In our experiments prenatal EGCG treatment of wild type mice did not induce significant alterations of the levels of synaptic markers or of NOR memory.

Therefore, controlled correction of active DYRK1A levels from prenatal through adult stages with a dietary complement containing a moderately (IC50 = 0.3 μM) potent drug such as EGCG might be the appropriate choice for a treatment to adequately balance risks and benefits.

## Materials and Methods

### Experimental mice

Mice carrying the murine BAC containing one copy of Dyrk1A (mBACtgDyrk1a) were maintained on a C57BL/6J background and genotyped as described^62^. Dp(16)1Yey mice were maintained on a C57Bl/6J background and genotyped as described^62^. Mice were group housed in standard cages with access to food and water ad libitum, under a controlled environment (20 ± 1 °C, 60% humidity) with a 12-h light/dark cycle.

All experiments were conducted in accordance with the ethical standards of French and European regulations (European Communities Council Directive, 86/609/EEC). For molecular analyses authorization from the French Ministry of Agriculture was granted to perform research and experiments on animals (authorization number 75–369), and the study was approved by the local ethical committee (Univ Paris-Diderot). For behavioural analyses the experimental procedures were approved by the local ethical committee Com’Eth under accreditation number 2012-069 with Y.H. as the principal investigator in this study (accreditation 67-369). Mice were fed standard laboratory diet (CRM,SpecialDietsServices, Dietex, FranceUsine) or food pellets containing 600 mg/kg MGTE (decaffeinated LifeExtension extract, containing 95% polyphenols), corresponding to a daily dose of 50 mg/kg EGCG for a 25 g mouse eating 5 g per day. (Safe-diets, Augny, France). HPLC analysis of an MGTE sample revealed a content of 38% EGCG, 9% EGC and 8.5% ECG and minor catechins. Number of mice and suffering were minimized as possible. Mice carrying the murine BAC containing one copy of Dyrk1A (mBACtgDyrk1a) were maintained on a C57BL/6J background and genotyped as described^62^. Dp(16)1Yey mice were maintained on a C57Bl/6J background and genotyped as described. After weaning mice were housed by 4 or 5. Food consumption was similar for wildtype and transgenic animals. For ECGC treatment, T1 treatment started at mating and continued through weaning (at 21 days). T2 treatment started at mating and continued until 90 days. T3 treatment started at P60 and continued until P90.

### Immunohistochemistry

Serial sagittal brain cryosections (50 µm) were immunohistochemically stained with GAD67 (Millipore MAB5406) and NeuN (Millipore ABN 78) antibodies. Neuronal and interneuronal cell densities were assessed on NeuN, and GAD67-stained sagittal serial sections (inter-section interval of 50 μm) (from Lateral 1,40 to 1,80 mm) with the optical fractionator probe of StereoInvestigator (MicroBrightField). The optical fractionator is a combination of the optical dissector, a three-dimensional probe used for cell counting and the fractionator random systematic sampling^63^.

Preliminary cell count was performed on the regions of interest (ROI) to determine the most suitable surface and number of counting frames resulting in coefficient of errors of Scheaffer and Gurndersen (m = 1) less than 0.05. Once these parameters were defined, cell counting was performed in a random systematic fashion using the optical fractionator with a dissector height of 40 μm and a guard zone of 5 μm. Neuronal and interneuronal density was estimated in Stratum Radiatum of the hippocampus using NeuN- and GAD67-stained serial sections using the following counting frame and grid sizes (counting frame 50 × 50 μm^2^; grid size 80 × 80 μm^2^). Both neuronal and interneuronal cells densities were defined using NeuN and GAD67-stained sections and the morphological criteria described by Gittins and Harrison (2004) were applied to differentiate these two cell populations. To avoid shrinkage related errors only cell/cell ratio of densities were compared. To illustrate cell density, images of Neun and GAD67 staining were captured with a Leica SP8 confocal microscope (Leica) Fig. 1.

### Immunoblotting

Immunoblotting was performed following standard slot blot protocols after testing antibody specificity by western blotting (Fig. 2 in Supplementary Methods). Antibodies are listed in Supplements. Digitized images of immunoblots were obtained using a LAS-3000 imaging system (Fuji Photo Film Co. Ltd.), and densitometry measurements were collected with an image analyzer (UnScan It software, Silk Scientific Inc.). Normalization was performed relative to total protein levels using Ponceau staining.

### Behavioural studies

For all behavioural experiments, Dp(16)1Yey mice were housed in specific pathogen-free conditions with ad libitum access to food and water. Mice were kept on 12-h light/dark cycles (lights on at 7:00 AM), and tests were conducted from 9:00 AM–4:00 PM. Only animals from litters containing a minimum of two male pups were selected for experimental groups. After weaning, male mice were gathered by litters in the same cage. Animals were transferred to the experimental room 30 min before each experimental test. Behavioural experimenters were blinded to genetic status and treatment group of the animals.

The Y-maze consisted of three arms (57 cm long × 17 cm wide × 35 cm high) in transparent plexiglass, and assembled at 120° angles. The Y-maze was placed 70 cm above the floor and was surrounded by visual cues (e.g., posters) outside of the maze. The room was illuminated by a desk lamp to maintain an intensity of 10 ± 3 Lux throughout the entire maze. A video camera suspended above the Y-maze was used to record arm entries.

The animal was placed at the center of the maze and allowed to move freely for a 10-min session. The number of arm entries was recorded during the first 5-min period and during the entire test. The number of alternations, which was defined as a successful entry into the three arms on overlapping triplet sets, was then calculated. The percent of successful alternations per possible alternations during the first 5-min period and during the entire test was calculated. The mean and standard error of the mean were calculated for each data group. Differences between groups were analyzed by a two-way ANOVA and a Student’s t-test. Statistical significance was considered at p < 0.05.

The novel object recognition test apparatus consisted of a white circular arena (diameter = 55 cm) placed in a dimly lit testing room (60 lux). On the first day of testing, mice were placed in the empty arena for 30 min for habituation to the apparatus and test room. On the second day of testing, mice were subjected to an acquisition trial, during which they were free to explore two identical objects for 10 min. Mice were individually placed in the presence of an object (marble or die) placed 10 cm away from one of the box corners. Exploration time of the object (when the animal’s snout was directed towards the object at a distance of ≤1 cm) was recorded.

After this acquisition phase, mice returned to their home cage for a 24-h retention interval. To test memory on the third day, one familiar object (i.e., already experienced during the acquisition phase) and one novel object were placed in the apparatus, and mice were free to explore the two objects for 12 min. Familiar objects and novel objects were placed at a distance 10 cm from the boder of the arena (distance between the two objects = ~27 cm), and exploration time of objects was recorded. Recognition index (RI) was defined as RI = (exploration timenovel objects − exploration timefamiliar objects)/(exploration timenovel objects + exploration timefamiliar objects). All mice that did not explore the first object for more than 3 seconds (below 3 seconds mice cannot memorize objects) during the acquisition trial were excluded from analysis. Analysis of exploration time was performed during 12 min. Between trials and subjects, different objects were cleaned with 70% ethanol to reduce olfactory cues. For each mouse, objects are randomly assigned as either familiar or novel to eliminate any effect due to spontaneous preference for an object. Location of the novel object (left or right) was counterbalanced between groups.

During all sessions, mice were monitored using a video tracking system (Ethovision, Wageningen, The Netherlands), which recorded total distance travelled and velocity. Object exploration was manually scored and defined as orientation of the nose to the object at a distance of <1 cm. For the retention phase, percent of time exploring familiar versus novel objects was calculated to assess memory performance.

### Statistical analysis

Differences between two groups were assessed with a two-tailed t-test. Two-Way ANOVA, followed by Holm–Sidak multiple comparison procedure, were used to compare data belonging to three groups or more than three groups. Level of significance was p < 0.05, unless otherwise specified. For results of behavioural experiments Kruskal Wallis analyses were also performed. All graphs were plotted as mean ± SEM. All statistical analyses were performed using GraphPad6 software package.

## Supplementary information

Dataset 1
