# Supplementary material for: Prenatal treatment with EGCG enriched green tea extract rescues GAD67 related developmental and cognitive defects in Down syndrome mouse models
Source: Sci Rep. 2019 Mar 8;9:3914. doi: 10.1038/s41598-019-40328-9 (PMC6408590; doi:10.1038/s41598-019-40328-9)
Supplement: Supplementary file 1 — Dataset 1 [file 41598_2019_40328_MOESM1_ESM.docx]

**Prenatal treatment with EGCG enriched green tea extract rescues GAD67 related developmental and cognitive defects in Down syndrome mouse models**

Benoit Souchet^1,9^, Arnaud Duchon^2,3,4,5,9^, Yuchen Gu^1^, Julien Dairou^6^, Claire Chevalier^2,3,4,5^, Fabrice Daubigney^1^, Valérie Nalesso^2,3,4,5^, Nicole Créau^1^, Yuejin Yu^7^, Nathalie Janel^1,7^, Yann Herault^2,3,4,5,10^§, Jean Maurice Delabar^1,8,10^§

^1^Université Paris-Diderot, Sorbonne Paris Cité, Adaptive Functional Biology, National Centre for Scientific Research (CNRS), UMR 8251, Paris, France

^2^Institut Génétique Biologie Moléculaire Cellulaire, CNRS, French National Institute of Health and Medical Research (INSERM), UMR 7104, UMR 964, Illkirch, France

^2^Institut de Génétique et de Biologie Moléculaire et Cellulaire, Université de Strasbourg, 1 rue Laurent Fries, 67404 Illkirch, France

^3^CNRS, UMR 7104, Illkirch, France

^4^INSERM, U964, Illkirch, France

^5^Université de Strasbourg, 1 rue Laurent Fries, 67404 Illkirch, France

^6^CNRS, UMR 8601, Laboratoire de Chimie et Biochimie Pharmacologiques et Toxicologiques, Université Paris Descartes-Sorbonne Paris Cité, 75270, Paris, France

^7^Children's Guild Foundation Down Syndrome Research Program, Department of Cancer Genetics, Roswell Park Cancer Institute, Elm and Carlton Streets, Buffalo, NY 14263, USA

^8^INSERM U 1127, CNRS UMR 7225, Sorbonne Universités, UPMC Univ Paris 06 UMR S 1127, Institut du Cerveau et la Moelle épinière, ICM, Paris, France; Brain & Spine Institute (ICM) CNRS UMR7225, INSERM UMRS 975, Paris, France

^9^ These authors contributed equally to this work.

^10^ These authors jointly directed this work.

§ Correspondence should be addressed to YH ([Herault@igbmc.fr](mailto:Herault@igbmc.fr)) for behavioral analysis and to JMD ([jeanmaurice.delabar@icm-institute.org](mailto:jeanmaurice.delabar@icm-institute.org) ) for molecular analysis.

**I Supplementary information and methods**

**HPLC methods**

**Quantification of catechins in green tea extracts**

Analysis of catechins by HPLC was performed as described by Lee et al (Cancer Epidemio Biomarkers Prevention 1995;4:393-399).

One mg of MGTE sample was solubilized in 10ml of bidistillated water at room temperature. Two hundred µL of this solution was extracted with fivefold ethyl acetate twice. The combined ethyl acetate solutions were evaporated to dryness in a SpeedVac concentrator and then were reconstituted in 100 µl of methanol/water (1:1, v/v). Extracted metabolites were stored at -80°C until HPLC analysis.

The HPLC analysis was done with the coulochem electrode array detection system (ESA 5600). The C18 reverse phase column (250*4.6 mm, particle size 5µm) was eluted at 40°C with a linear gradient from 96% buffer A (30 mM NaH2PO4 buffer containing 0.12% of tetrahydrofuran (pH 3.35)) and 4% buffer B (30 mM NaH2PO4 buffer containing 40% acetonitrile and 6.65% tetrahydnofuran (pH 3.45)) to 76% buffer A and 24% buffer B in 24 mm at a flow rate of 1 mL/min. Then the gradient was changed linearly to 5% buffer A and 95% buffer B from 24 to 35 min and maintained at 5% buffer A and 95% buffer B until 42 min. Then, the gradient was changed back to 96% buffer A and 4% buffer B for the analysis of the next sample. The eluent was monitored by the electrochemical detector with potential settings at -90, -10, 70, 150, 230, 310, 390, and 470 mV, and 8 chromatograms were obtained simultaneously. The products were quantified by integration of the peak absorbance area, employing a calibration curve established with various known concentrations of catechins.

**Figure 1:** HPLC calibration curves for Catechin, EGC and EGCG

**Quantification of EGCG in biological samples**

The biological samples levels of total (sulfatase/glucuronidase-treated) catechins were analyzed as previously reported (Chen et al. 1997). One hundred microliters of plasma (or brains extracts) samples were thawed at room temperature and 50 µL of 0.4 M sodium phosphate buffer (pH 7.4) were added. The sample was mixed with 10 µL of b-glucuronidase (250 units) and 10 µL of sulfatase (10 units) and then incubated at 37 C for 45 min. Then this solution was extracted with fivefold ethyl acetate twice. The combined ethyl acetate solutions were evaporated to dryness in a SpeedVac concentrator and then were reconstituted in 100 µl of methanol/water (1:1, v/v). Extracted metabolites were stored at -80°C until HPLC analysis.

**DYRK1A inhibitory activity assessment**

Kinetic analysis and inhibition of Dyrk1a as described by Bui et al (Anal biochem 2014; 449;172-178) by HPLC-based separation and quantitation of a fluorescein-labeled peptide substrate and its phosphorylated product. The purity and identity of the fluorescein-labeled peptide substrate (FAM–peptide was synthesized by Genosphere (Paris, France) was initially assessed by reverse-phase HPLC (Prominence Shimadzu UFLC [ultra-fast liquid chromatography] system interfaced with LabSolutions software). Samples were injected into an Ascentis Express C8 column (length = 150 mm, internal diameter = 4.6 mm, particle size = 2.7 lm) at 40°C. The mobile phase used for the separation consisted of two eluents; solvent A was water with 0.12% trifluoroacetic acid (TFA), and solvent B was acetonitrile with 0.12% TFA. Compounds were separated by an isocratic flow (85% A/15% B) rate of 1.5 ml/min. The products were monitored by fluorescence emission (lambda = 530 nm) after excitation at lambda = 485 nm and quantified by integration of the peak absorbance area, employing a calibration curve established with various known concentrations of peptides. Assays were performed in a 96-well ELISA plate in a total volume of 50 µl consisting of kinase buffer (50 mM Tris–HCl, 10 mM DTT, and 5 mM MgCl2), ATP (up to 1000 µM), FAM–peptide substrate (up to 60 µM), and purified Dyrk1a (up to 0.5 ng). Briefly, samples containing the enzyme were preincubated with peptide substrate at 37°C for 1 min, and the reaction was started by the addition of ATP. At different time points (up to 30 min), 50 µl of HClO4 (15% in water) was added to stop the reaction, and 20 µl was automatically injected into the HPLC column.

**Antibodies**

**Figure 2: S**ignals obtained on western immunoblotting of protein extracts from A: wild type (Wt) cerebellum (Crb) and cortex (Ctx) for DYRK1A; B: wt and tg ( mBACtgDyrk1a) hippocampus for GAD67; C: wt hippocampus for GAD65; D: wt cortex for VGAT1; E: wt cortex for PSD95; F: wt and tg cortex for VGLUT1; G: wt and tg cortex for NR2A; H: wt cortex for NR1; I: wt cortex for GLUR2.

**II Supplementary Data**

**Treatment effects on protein levels**

**Quantification of GAD67+ neurons in colliculus**

**Figure 3**: Quantification of GAD67+ neuron fraction of NeuN+ neurons in immunohistochemically stained sections of colliculus of control (n = 5) wildtype (WT) and trisomic (n=5) Dp16(1)Yey (Ts) mice:
